# A Systematic Review of Reproductive Counseling in Cases of Parental Constitutional Reciprocal Translocation (9;22) Mimicking BCR-ABL1

**DOI:** 10.3389/fgene.2022.921910

**Published:** 2022-08-04

**Authors:** Zimeng Gao, Stephanie M. Rice, Sascha Wodoslawsky, Sara C. Long, Zi-Xuan Wang, Mehnoosh Torkzaban, Ana Milena Angarita Africano, Jinglan Liu, Huda B. Al-Kouatly

**Affiliations:** ^1^ Department of Obstetrics and Gynecology, Sidney Kimmel Medical College at Thomas Jefferson University, Philadelphia, PA, United States; ^2^ Department of Obstetrics and Gynecology, Division of Maternal-Fetal Medicine, Sidney Kimmel Medical College at Thomas Jefferson University, Philadelphia, PA, United States; ^3^ Sidney Kimmel Medical College at Thomas Jefferson University, Philadelphia, PA, United States; ^4^ Department of Pathology, Clinical Cytogenomics, Anatomy, and Cell Biology, Sidney Kimmel Medical College at Thomas Jefferson University, Philadelphia, PA, United States; ^5^ Department of Radiology, Sidney Kimmel Medical College at Thomas Jefferson University, Philadelphia, PA, United States

**Keywords:** translocation, chromosome aberrations, Philadelphia chromosome, chromosome breakpoints, reproductive counseling

## Abstract

We aim to determine the spectrum of cytogenetic abnormalities and outcomes in unbalanced offspring of asymptomatic constitutional balanced t(9;22) carriers through a systematic literature review. We also include a case of a constitutional balanced t(9;22) carrier from our institution. Among the 16 balanced t(9;22) carriers in our review, 13 were maternal and 3 were paternal. Of the 15 unbalanced translocation cases identified, 13 were live births, one was a missed abortion, and one resulted in pregnancy termination. The spectrum of established syndromes reported among the live births was the following: trisomy 9p syndrome (6/13), dual trisomy 9p and DiGeorge syndrome (3/13), dual 9q subtelomere deletion syndrome and DiGeorge syndrome (1/13), 9q subtelomere deletion syndrome (1/13), and DiGeorge syndrome (1/13). One unbalanced case did not have a reported syndrome. The phenotype of the unbalanced cases included cardiac abnormalities (5/13), neurological findings (7/13), intellectual disability (6/10), urogenital anomalies (3/13), respiratory or immune dysfunction (3/13), and facial or skeletal dysmorphias (13/13). Any constitutional balanced reciprocal t(9;22) carrier should be counseled regarding the increased risk of having a child with an unbalanced translocation, the spectrum of possible cytogenetic abnormalities, and predicted clinical phenotype for the unbalanced derivative.

## Introduction

Chromosomal translocations are structural abnormalities characterized by the exchange of chromosomal segments between nonhomologous and homologous chromosomes. Balanced translocations refer to instances of chromosomal rearrangement without apparent loss in genetic material and full functionality, while unbalanced translocations result in an overall genetic gain or loss (Morin et al., 2017). Balanced or unbalanced structural translocations have a frequency of 1.7% in the general population (Paththinige et al., 2019). Apparently balanced chromosomal arrangements have an estimated frequency of 0.5% in newborns, of which 14% are *de novo* (Jacobs et al., 1992).

Apparently constitutional balanced translocations associated with normal phenotypes can be transmitted through generations without major adverse effects, except for an increased risk of reproductive loss or abnormal phenotypes in offspring (Gribble et al., 2005). Phenotypically normal carriers of balanced translocations are at a higher risk of producing offspring with unbalanced translocations with potential outcomes of developmental delay, congenital cardiac defects, urogenital anomalies, respiratory or immune dysfunctions, or facial or skeletal dysmorphias (Ganguly et al., 2011). These carriers of balanced translocations are frequently identified only after recurrences of unbalanced offspring within families. If an abnormal phenotype is identified in a balanced translocation carrier, chromosomal microarray analysis (CMA) or next-generation sequencing (NGS) may be performed to detect possible copy-number variation (CNV) at breakpoints of the translocation (Weckselblatt and Rudd, 2015). Submicroscopic deletions or duplications can be seen in 46% of individuals with an abnormal phenotype who carry a *de novo* apparently balanced chromosomal rearrangement, and in 25% of affected individuals with an inherited apparently balanced chromosomal rearrangement (Tabet et al., 2015).

Several uncommon constitutional balanced translocations have been reported, which include: t(8;22)(q24.13;q11.21), t(4;8)(p16;p23), t(4;11)(p16.2;p15.4), t(8;12)(p23.1;p13.31), and t(9;22)(q34;q11.2). These translocations result from nonallelic homologous recombination through breaks in adenine and thymine residue-rich (AT-rich) repeats repaired by nonhomologous end-joining (Czuchlewski et al., 2011; Ou et al., 2011). Some balanced translocations such as the Philadelphia chromosome, t(9;22)(q34;q11.2), may be postnatally acquired. This acquired translocation is characterized by a *BCR-ABL1* gene fusion responsible for chronic myeloid leukemia (CML), certain subtypes of acute myeloid leukemia (AML), and acute lymphoblastic leukemia (ALL).

In our systematic literature review of constitutional reciprocal t(9;22) carriers, we describe the genetic and phenotypic characteristics of their unbalanced offspring. Our review was inspired by a case at our institution, in which a patient with a history of recurrent pregnancy loss carried a constitutional balanced t(9,22)(q34;q11.2). This uncommon translocation, which mimics the somatic balanced translocation seen in CML, AML, and ALL subtypes, has only been reported twice in prior literature (McGoey and Lacassie, 2009; Czuchlewski et al., 2011). In this review, we investigate how this translocation pertains to her pregnancies.

## Case

A 31-year-old G3P0020 woman of Indian descent presented for prenatal genetic counseling with her partner at 13w5d gestation. The patient has a past medical history of pulmonic stenosis and hidradenitis suppurativa. Her partner is also of Indian descent and a self-reported heterozygote for Factor V Leiden identified through direct-to-consumer genetic testing. The couple had previously experienced two early first trimester pregnancy losses, and genetic testing was not performed on the couple or the products of conception in either pregnancy loss. The couple underwent a workup for recurrent pregnancy loss at a local reproductive endocrinology and infertility clinic.

**FIGURE 1:**
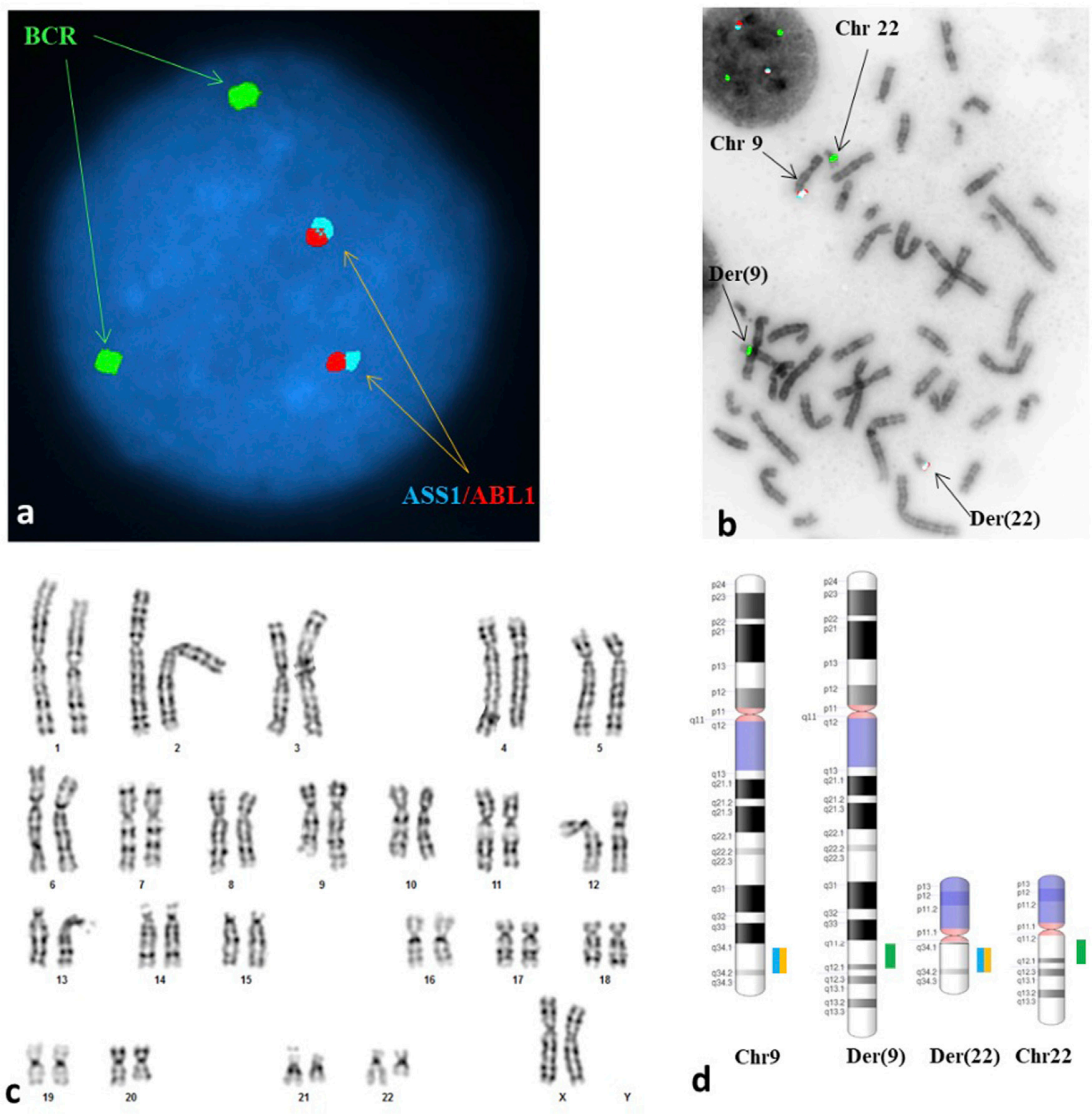
A reciprocal translocation of t(9;22)(q34;q11.2) involving neither the ABL1 locus at 9q34 nor the BCR at 22q11.2 was seen in each of the metaphases analyzed. **(A)** Interphase FISH study showed a normal signal pattern; probes for the ASS1*, ABL1 and BCR were labeled in aqua, red, and green, respectively. **(B)** Metaphase FISH study showed an abnormal hybridization pattern with no translocation of BCR/ABL1 observed. The arrows are pointing to the derivative chromosome 9 [der(9)] carrying a signal for BCR but no signals for the ASS1 and ABL1, the derivative chromosome 22 [der(22)] carrying signals for the ASS1 and ABL1 but no signal for the BCR, the normal chromosome 9 [chr 9] and the normal chromosome 22 [chr 22]. **(C)** Karyogram exhibited an apparently balanced reciprocal translocation between the long arm of chromosome 9 at 9q34 and the short arm of chromosome 22 at 22q11.2, with morphology indistinguishable from the Philadelphia translocation. **(D)** A schematic illustration of the normal copies of chromosomes 9 and 22 as well as the derivative chromosomes 9 and 22 resulted from the reciprocal translocation. The breakpoints at each derivative chromosome involved were located at the vicinity but proximal to the loci involved in the Philadelphia translocation. *ASS1 is a control probe at 9q34 overlapping the ABL1 probe.

The patient’s partner had a normal male karyogram (46, XY), while the patient was found to have an apparently balanced reciprocal translocation between chromosomes 9 and 22 [46,XX,t(9;22)(q34;q11.2)] (Figure 1C; Appendix B). The breakpoints of her constitutional translocation are similar to those of the somatic balanced translocation t(9,22)(q34;q11.2) observed in CML and certain AML and ALL subtypes. Given these break point similarities, fluorescent *in situ* hybridization (FISH) was performed on both interphase nuclei and metaphase cells (Appendix B). The interphase FISH study showed no *BCR-ABL1* fusion (Figure 1A), while metaphase FISH demonstrated a translocation between the long arms of chromosomes 9 and 22 at 9q34 and 22q11.2, respectively, without involvement of the *ABL1* and *BCR* genes (Figures 1B, D). Despite having seemingly identical chromosomal alterations by conventional cytogenetic analysis, the negative FISH results confirmed that our patient did not have a pathogenic t(9;22)(9q34;22q11.2) that results in a *BCR-ABL1* chimeric product.

Additionally, no evidence of leukemic process was identified on complete blood count (CBC). Further workup with reverse transcription-polymerase chain reaction (RT-PCR) was recommended to quantitatively detect t(9;22) *BCR-ABL1* transcripts. The absence of transcripts would rule out the possibility of a cryptic translocation that could result in leukemia, though unlikely given the patient’s normal CBC. The patient declined further workup with RT-PCR.

The patient’s nuchal translucency ultrasound and first trimester sequential screen were normal. After reviewing her karyotype and discussing the increased risk of an unbalanced translocation in her fetus, the patient elected amniocentesis. The results of the amniocentesis revealed a normal male fetal karyotype (46,XY) with no clinically significant numerical or structural chromosome abnormalities. Chromosome 9 and 22 homologs appeared normal at the 450-band resolution of karyotyping. Additionally, the CMA was consistent with a normal male fetus without clinically significant CNV or copy neutral regions detected at a resolution of 50 Kb or higher (Appendix B). Finally, both her amniotic fluid alpha-fetoprotein (AF-AFP) testing and limited fetal anatomy ultrasound completed at the time of amniocentesis were normal.

An echocardiogram was performed to evaluate the patient’s pulmonic stenosis at 18 weeks gestation, which indicated an increase in trans-pulmonic gradient as compared to her cardiac MRI images from two years prior. This increase, though still in the mild range, was attributed to the high-flow state in pregnancy. A repeat echocardiogram was completed at 30 weeks gestation, which indicated no significant changes since the prior study at 18 weeks.

The patient underwent parental CMA to determine the etiology of her pulmonic valve stenosis by evaluating sub-microscopic CNV(s) not detectable by standard karyotyping. Her CMA results were normal, making the translocation an unlikely cause of her cardiac finding. However, the normal result does not exclude the presence of a CNV <50 Kb or the possibility of a monogenic disorder as the cause of the stenosis. The patient declined single gene testing.

The remainder of the pregnancy was uneventful except for the identification of a fetal right pelvic kidney with normal renal parenchyma diagnosed at 20 weeks gestation by anatomy ultrasound. The patient had a spontaneous vaginal delivery of a live male infant weighing 3.37 kg at 40w3d. Apgar scores were 8 and 9 at 1 and 5 minutes, respectively. The neonate was discharged on the second day of life with no complications. He is currently in good health at 17 months of age without any health consequences from his right pelvic kidney.

## Materials and Methods

**FIGURE 2:**
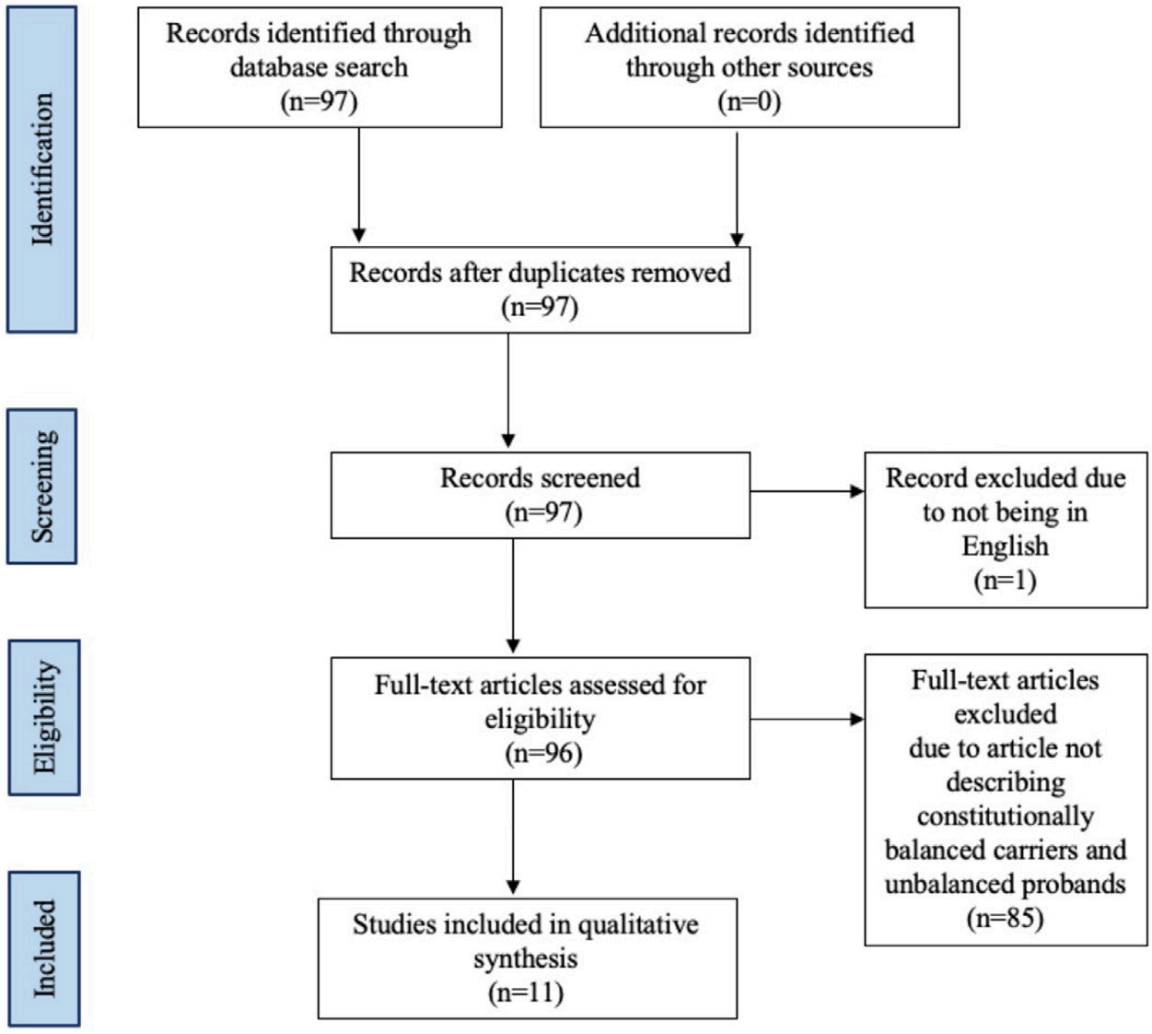
Flow diagram of studies identified in the systematic review.

The literature review was conducted using PubMed and Scopus from database inception to 10 October 2021. Articles discussing constitutional translocations of chromosomes 9 and 22 were identified without limitations on study date or type. Only literature in English were included (Figure 2). The following search terms were used to identify extant literature: 9;22, (9;22)(q34;q11.2), t(9;22)(q34;11.2), translocation, congenital, constitutional, and inherited (Appendix A). The reference list of each article identified was reviewed for additional articles. Data extraction from the articles was performed according to PRISMA guidelines (Figure 2) (Page et al., 2021). We refer to unbalanced t(9;22) offspring of balanced t(9;22) carriers as “cases” in our review.

## Results

A total of 11 studies including 15 cases of unbalanced translocations from a balanced t(9;22) carrier parent met our inclusion criteria. Table 1 summarizes the cytogenetics of these unbalanced cases. One balanced translocation carrier had primary ovarian insufficiency and thus no pregnancies reported (Peterson et al., 2019). Twelve cases were maternal in origin, while three were paternal. Of the 15 pregnancies included in our literature review, 13 resulted in live births, one resulted in a missed abortion, and one pregnancy was terminated (Tables 2, 3). Of the 13 live births, three cases passed away from cardiopulmonary complications within six months of birth and 10 cases survived to adulthood or the time of publication with ages ranging from 22 months to 35 years. Pregnancy complications were reported for five of the 13 live births (Table 2). Two neonates were small for gestational age, one of whom also had polyhydramnios and required cesarean delivery secondary to fetal distress. One neonate had an irregular fetal heart rate detected at 33 weeks gestation. Two neonates had uneventful prenatal courses.

**TABLE 1:** Summary of cytogenetic findings of unbalanced t(9;22) cases.

Unbalanced cytogenetic finding	Number of cases (%)
Trisomy 9p syndrome	6 (40)
Dual trisomy 9p syndrome and DiGeorge syndrome	3 (20)
Dual 9q subtelomere deletion syndrome and DiGeorge syndrome	1 (6.7)
9q subtelomere deletion syndrome	1 (6.7)
DiGeorge syndrome	1 (6.7)
Other^a^	3 (20)
Total number of cases	15

a Unbalanced cases without reported cytogenetic findings.

**TABLE 2:** Pregnancy outcomes of constitutional reciprocal t(9;22) carriers.

Case	Balanced parental carrier	Case karyotype	Pregnancy complications	Cytogenetic outcomes of all carrier pregnancies	Miscarriages	Family history
Blank et al. (1975) ^a^						
Case 1	Maternal	46,XX,+der(9)t(9;22)(q13;q11),-22	None reported	1 trisomy 9p syndrome	2, did not specify gestational age (karyotype not done)	Family history of recurrent pregnancy loss, recurrent familial trisomy 9p syndrome
				1 balanced translocation		
				1 deceased (unknown karyotype and phenotype)		
Case 2	Maternal	46,XX,+der(9)t(9;22)(q13;q11),−22	None reported	2 trisomy 9p syndromes	1, did not specify gestational age (karyotype not done)	Family history of recurrent pregnancy loss, recurrent familial trisomy 9p syndrome
				2 balanced translocations		
				1 normal karyotype		
Case 3	Maternal	46,XX,+der(9)t(9;22)(q13;q11),−22	None reported	2 trisomy 9p syndromes	1, did not specify gestational age (karyotype not done)	Family history of recurrent pregnancy loss, recurrent familial trisomy 9p syndrome

				2 balanced translocations 1 normal karyotype		
Case 4	Maternal	46,XY,+der(9)t(9;22)(q13;q11),−22	None reported	1 trisomy 9p syndrome	None reported	Family history of recurrent pregnancy loss, recurrent familial trisomy 9p syndrome


Pivnick et al. (1990) ^b^						
Case 1	Maternal	46,XX,−22,+der(9)t(9;22)(q22;q11.2)	Cesarean section for fetal distress, polyhydramnios, small for gestational age (birth weight 2000 g at 38 weeks)	1 dual trisomy 9p syndrome and DiGeorge syndrome	None	Family history not reported

				1 unbalanced translocation: der(9)t(9;22)(q22;q11.2),−22		
Case 2	Maternal	46,XY,−22,+der(9)t(9;22)(q22;q11.2)	Prenatal diagnosis with CVS followed by pregnancy termination	1 dual trisomy 9p syndrome and DiGeorge syndrome	None	Family history not reported

				1 unbalanced translocation: der(9)t(9;22)(q22;q11.2),−22		
El-Fouly et al. (1991)						
	Maternal	46,XX,−22,+der(9)t(9;22)(q21.13;q12.1)	Small for gestational age (birth weight 2298 g at 38.5 weeks)	1 dual trisomy 9p syndrome and DiGeorge syndrome	None	No reported miscarriages or anomalies similar to those observed in the case

				2 phenotypically normal (karyotype not done)		
Tihy et al. (2000)						
	Maternal	47,XX,+der(9)t(9;22)(q12;p11)	None reported	1 trisomy 9p syndrome	2, gestational age not specified (karyotype not done)	Family history not reported

				2 phenotypically normal (karyotype not done)		
Komatsu et al. (2001)						
	Paternal	46,XX,+der(9)t(9;22)(q12;q11.23),−22	None reported	1 dual trisomy 9p syndrome and DiGeorge syndrome	1, first trimester (karyotype not done)	No family history of clinical findings consistent with those of the case



Sanger et al. (2005) ^c^						
Case 1	Maternal	46,XY,der(9)t(9;22)(q34.3;p11.2)	Irregular fetal heart rate detected at 33 weeks	1 9q subtelomere deletion syndrome	1, 6 weeks gestation (karyotype not done)	Maternal grandparent had a stillbirth at 8 months due to presumed cord accident. Maternal great-aunt (Case 2) and one of her daughters both have learning disabilities
				1 phenotypically normal male with a balanced translocation		
Case 2	Paternal	46,XX,der(22)t(9;22)(q34.3;p11.2)	None reported	1 unbalanced translocation	None reported	Her brother had a stillbirth at 8 months due to presumed cord accident. One of her daughters has learning disabilities^d^
				1 balanced translocation		
				1 phenotypically normal with unknown karyotype (unable to conceive)		
McGoey and Lacassie (2009)						
	Paternal	45,XX,der(9)t(9;22)(q34.3;q11.2),−22	Normal prenatal course and normal prenatal ultrasounds	1 dual 9q subtelomere deletion syndrome and DiGeorge syndrome	None	Paternal family history of muscular dystrophy


Shuib et al. (2009)						
	Maternal	45,XX,der(9)t(9;22)(p23;q11.2),−22	None reported	1 DiGeorge syndrome	None reported	Family history not reported

Czuchlewski et al. (2011)						
	Maternal	45,XX,der(9)t(9;22)(q34;q11.2),−22	Missed abortion	1 unbalanced translocation	3, first trimester (karyotype not done)	Family history not reported

Bouhjar et al. (2011)						
	Maternal	47,XY,+der(22)t(9;22)(p13.1;q11)	Uneventful pregnancy	1 trisomy 9p syndrome	3, all after 2 months of gestation (karyotype not done)	Family history not reported
	
				3 phenotypically normal brothers with unknown karyotype		
Gao et al. (2022) - Our case						
	Maternal	Normal karyotype	Fetal right pelvic kidney	Normal karyotype and microarray	2, gestational ages not specified (karyotype not done)	Maternal history of pulmonic stenosisPaternal family history of Factor V Leiden mutation

Abbreviations: CVS, chorionic villus sampling.

a For Blank et al. (1975) the four cases are reported within one family. Case 1 is the first cousin of Case 2 and 3. Case 2 and 3 are siblings. Case 4 is the nephew of Case 1.

b For Pivnick et al. (1990) the two cases are two pregnancies from the same patient.

c For Sanger et al. (2005) the two cases are reported within one family. Case 2 is the maternal great-aunt of Case 1.

d For Sanger et al. (2005) Case 2 has 3 children. The children did not have cytogenetic testing done; however, one of the daughters was reported as “slow,” which Case 2 was also described as being.

**TABLE 3:** Unbalanced translocation cases resulting from constitutional reciprocal t(9;22) carriers.

Case	Parental karyotype	Parental origin^a^	Sex GA at birth Birthweight (g)	Case karyotype	Recognizable phenotype reported	Case age at time of publication
Blank et al. (1975) ^a^						
Case 1	Balanced 46,XX,t(9;22)(q13;q11)	Maternal	Female NR	46,XX,+der(9)t(9;22)(q13;q11),-22	*Trisomy 9p syndrome:*	35 years old

			2800		Severe intellectual disability, marked kyphosis, epilepsy, macroglossia, small and/or dysplastic toes with dystrophic toenails	
Case 2	Balanced 46,XX,t(9;22)(q13;q11)	Maternal	Female	46,XX,+der(9)t(9;22)(q13;q11),-22	*Trisomy 9p syndrome:*	23 years old
			NR			
			3000		Severe intellectual disability, moderate kyphosis, eczema, bilateral keratosis, small and/or dysplastic toes with dystrophic toenails	
Case 3	Balanced 46,XX,t(9;22)(q13;q11)	Maternal	Female	46,XX,+der(9)t(9;22)(q13;q11),-22	*Trisomy 9p syndrome:*	19 years old
			NR			
			3500		Severe intellectual disability, hirsute back and forearms, diastasis recti, syndactyly of all fingers, small and/or dysplastic toes with dystrophic toenails	
Case 4	Balanced 46,XX,t(9;22)(q13;q11)	Maternal	Male	46,XY,+der(9)t(9;22)(q13;q11),-22	*Trisomy 9p syndrome:*	7 years old
			NR			
			3700		Severe intellectual disability, marked kyphosis, epilepsy, macroglossia, small and/or dysplastic toes	
Pivnick et al. (1990) ^b^						
Case 1	Balanced 46,XX,t(9;22)(q22;q11.2)	Maternal	Female	46,XX,-22,+der(9)t(9;22)(q22;q11.2)	*Trisomy 9p syndrome and DiGeorge syndrome:*	Neonatal mortality at 3 weeks of age due to cardiovascular complications
			38 weeks			
			2000		Facial dysmorphism, generalized hypotonia, multiple cardiac anomalies (patent ductus arteriosus, interrupted aortic arch [type B], misaligned ventricular septal defect, aortic stenosis), absent thymus and immune dysfunction, hypocalcemia, urogenital anomalies and hydronephrosis	
Case 2	Balanced 46,XX,t(9;22)(q22;q11.2)	Maternal	MaleNRNR	46,XY,-22,+der(9)t(9;22)(q22;q11.2)	None reported	Pregnancy termination after prenatal diagnosis by CVS, gestational age not reported


El-Fouly et al. (1991)						
	Balanced 46,XX,t(9;22)(q21.13;q12.1)	Maternal	Female 38.5 weeks 2298	46,XX,-22,+der(9)t(9;22)(q21.13;q12.1)	*Trisomy 9p syndrome and DiGeorge syndrome:*	Neonatal mortality at day 10 of life due to cardiopulmonary failure
	
					Microcephaly, facial dysmorphism, hypoplastic lungs, cardiac anomalies (type II truncus arteriosus, truncal valve stenosis of three abnormal cusps, a single carotid trunk, atrial and ventricular septal defects), hypoplastic thymus, multiple small accessory spleens	
Tihy et al. (2000)						
	Balanced 46,XX,t(9;22)(q12;p11)	Maternal	Female Term birth 2700	47,XX,+der(9)t(9;22)(q12;p11)	*Trisomy 9p syndrome:*	22 months old

					Psychomotor impairment, prenatal and postnatal growth restriction, microcephaly, brachycephaly, generalized hypotonia, strabismus, myopia, short neck, small hands and feet, and brachymesophalangy	
Komatsu et al. (2001)						
	Balanced 46,XY,t(9;22)(q12;q11.23)	Paternal	Female 40 weeks 2748	46,XX,+der(9)t(9;22)(q12;q11.23),-22	*Trisomy 9p syndrome and DiGeorge syndrome:*	Neonatal mortality at 6 months of age due to cardiopulmonary failure

					Facial dysmorphism, cardiac anomalies (truncus arteriosus type A2 with bilateral pulmonary arteries, misaligned ventricular septal defect with overriding truncus arteriosus, absence of patent ductus arteriosus), bilateral hydronephrosis, palatal anomaly, retrognathia, laryngeal hypotonia	
Sanger et al., (2005) ^d^						
Case 1	Balanced 46,XX,t(9;22)(q34.3;p11.2)	Maternal	Male 38 weeks 3440	46,XY,der(9)t(9;22)(q34.3;p11.2)	*9q subtelomere deletion syndrome:*	8 years old
					Facial dysmorphism, micrognathia, congenital heart disease (patent ductus arteriosus, ventricular septal defect, pulmonary valve stenosis)	
Case 2	Balanced 46,XY,t(9;22)(q34.3;p11.2)	Paternal	FemaleNRNR	46,XX,der(22)t(9;22)(q34.3;p11.2)	Mild facial dysmorphism, high arched palate, learning disability	Adult
	

McGoey and Lacassie (2009)						
	Balanced 46,XY,t(9;22)(q34.3;q11.2)	Paternal	Female 39 weeks 2880	45,XX,der(9)t(9;22)(q34.3;q11.2),-22	*9q subtelomere deletion syndrome and DiGeorge syndrome:*	Not reported
	
					Cardiac anomalies (ventral septal defect, patent ductus arteriosus, right ventricular hypertrophy, interrupted aortic arch), bilateral renal hypoplasia, generalized hypotonia, microbrachycephaly, micrognathia, prominent clitoris	
Shuib et al. (2009)						
	Balanced 46,XX,t(9;22)(p23;q11.2)	Maternal	FemaleNRNR	45,XX,der(9)t(9;22)(p23;q11.2),-22	*DiGeorge syndrome:*	8 years old
					Coarse facies, learning disability	

Czuchlewski et al. (2011)	Balanced 46,XX,t(9;22)(q34;q11.2)	Maternal	FemaleNRNR	45,XX,der(9)t(9;22)(q34;q11.2),-22	None reported^e^	Missed abortion, gestational age not reported

	
Bouhjar et al. (2011)						
	Balanced 46,XX,t(9;22)(p13.1;q11)	Maternal	Male Term birth 4000	47,XY,+der(22)t(9;22)(p13.1;q11)	*Trisomy 9p syndrome*:Facial dysmorphism, short fingers and toes, normal intellectual development, asthma, recurrent respiratory infections	10 years old

Abbreviations: GA, gestational age; NR, none reported; CVS, chorionic villus sampling.

a Denotes origin of balanced translocation for unbalanced karyotype in cases.

b For Blank et al. (1975), the four cases are reported within one family. Case 1 is the first cousin of Case 2 and 3. Case 2 and 3 are siblings. Case 4 is the nephew of Case 1.

c For Pivnick et al. (1990) the two cases are two pregnancies from the same patient.

d For Sanger et al. (2005) the two cases are reported within one family. Case 2 is the maternal great-aunt of Case 1.

e Denotes karyotype performed on products of conception of missed abortion, with the phenotype not reported.

### Phenotype of Unbalanced Cases

The phenotypes of the unbalanced cases include an array of features such as cardiac abnormalities, neurological findings, intellectual disability, urogenital abnormalities, respiratory and immune dysfunctions, and facial or skeletal dysmorphias (Table 3, Table 4). Only a few of these findings were found in isolation.

**TABLE 4:** Summary of phenotypes of unbalanced t(9;22) cases.

Phenotypic outcome	Number of cases reported	Cases affected (%)
Cardiac abnormalities	5	38
Neurologic findings	7	54
Intellectual disability^a^	6	60
Urogenital anomalies	3	23
Respiratory or immune dysfunctions	3	23
Facial or skeletal dysmorphias	13	100
Total number of cases	13^b^	

a Three live births were not able to be intellectually assessed due to neonatal mortality at less than 6 months of age, making the denominator 10 instead of 13.

b Two reported cases could not be phenotypically assessed as one was a missed abortion and one was a pregnancy termination.

### Cardiac Abnormalities

Congenital heart disease was found in five of 13 live births. Of these five cases, three passed away from cardiopulmonary complications within six months of birth, one case was eight years old at time of report, and one case did not have a reported clinical outcome. Of these same five cases, three had a dual diagnosis of trisomy 9p syndrome and DiGeorge syndrome (22q11.2 deletion syndrome), one had 9q subtelomere deletion syndrome, and one had a dual diagnosis of 9q subtelomere deletion syndrome and DiGeorge syndrome (Table 3). All three neonatal deaths from cardiac abnormalities had dual diagnoses of trisomy 9p and DiGeorge syndrome. All eight unbalanced cases without significant cardiac abnormalities survived to adulthood or to the time of publication.

### Neurological Findings

Seven of 13 live births presented with neurological findings. Two cases had epilepsy (Blank et al., 1975), three had generalized hypotonia (Pivnick et al., 1990; Tihy et al., 2000; McGoey and Lacassie, 2009), and one had laryngeal hypotonia (Komatsu et al., 2001). El-Fouly, Tihy, and McGoey also describe microcephaly, brachycephaly, or a combination of both conditions in their publications (McGoey and Lacassie, 2009; Tihy et al., 2000; El-Fouly et al., 1991).

### Intellectual or Learning Disabilities

At least six of the 10 live births (60%) who were able to be cognitively assessed have some degree of intellectual or learning disability. Blank et al. (1975) reports four cases with severe intellectual disability. Sanger and Shuib each report one case with a learning disability (Sanger et al., 2005; Shuib et al., 2009).

### Urogenital Anomalies

Urogenital findings were reported in three of the 13 cases. Pivnick et al. (1990) described one case of hydronephrosis and hypoplastic and posteriorly fused flat labia majora, while Komatsu et al. (2001) reported bilateral hydronephrosis in one case. McGoey and Lacassie (2009) reported one case with bilateral renal hypoplasia, unilateral simple renal cysts, and a prominent clitoris.

### Respiratory and Immune Findings

Three of the 15 cases had pulmonary or immune dysfunction. One case reported in Pivnick et al. (1990) had an absent thymus with subsequent immune dysfunction. El-Fouly et al. (1991) reported one case with hypoplastic lungs and thymus, and Bouhjar et al. (2011) reported one case with asthma with recurrent respiratory infections.

### Facial or Skeletal Dysmorphic Findings

All unbalanced cases with reported phenotypes had some form of facial or skeletal dysmorphia. Seven of the 13 live births had facial dysmorphia, and four had malformations such as microbrachycephaly, microcephaly, micrognathia, retrognathia, and macroglossia (McGoey and Lacassie, 2009; Blank et al., 1975; Pivnick et al., 1990; Komatsu et al., 2001; El-Fouly et al., 1991; Sanger et al., 2005; Shuib et al., 2009; Bouhjar et al., 2011). Six cases had reported skeletal anomalies, all of which include small bones in the hands and feet. Blank et al. (1975) reports four cases with small and/or dysplastic toes, Tihy et al. (2000) reports one case of small hands and feet, and Bouhjar et al. (2011) reports one case of short fingers and toes.

### Genetic Family History of Balanced t(9;22) Carriers

Nine of the 15 balanced carriers (60%) had at least one prior miscarriage (Table 2). Recurrent pregnancy loss, defined as ≥2 pregnancy losses, occurred in five (33%) pregnancies. All but one miscarriage involved balanced translocations of maternal origin (Komatsu et al., 2001). Four of the balanced carriers, three of which were maternal and one of which was paternal, did not have any miscarriages. Additionally, the four family members in Blank et al. (1975) with trisomy 9p syndrome, including the parents, maternal grandparents, and first cousins of Case 1, reported several miscarriages. Three balanced carriers did not specify a history of miscarriage.

Eight of the 15 unbalanced cases included information on their siblings. Case 1 in Blank et al. (1975) had two siblings, one of whom had a balanced translocation, and the other resulted in an infant death without a reported cause or genetic workup. Case 2 and 3 in the same paper were siblings with three other siblings in the household, two of whom had the balanced translocation, while the other had a normal karyotype. The unbalanced cases described by El-Fouly et al. (1991) and Tihy et al. (2000) each have two siblings who are phenotypically normal with an unknown karyotype. Case 1 in Sanger et al. (2005) has one brother with a balanced translocation. Case 2 has two brothers, one with a balanced translocation and the other phenotypically normal with an unknown karyotype, but reported infertility. The cases in Bouhjar et al. (2011) had three total siblings, all of whom were phenotypically normal but had unknown karyotypes.

## Discussion

The apparently balanced translocations of healthy individuals can remain undetected until pregnancy when patients present with recurrent pregnancy loss or fetal congenital abnormalities (Gardner, 2004). Our patient, who only had a past medical history of mild pulmonic stenosis, sought reproductive counseling due to recurrent pregnancy loss and was diagnosed with a constitutional balanced t(9;22)(q34;q11.2). The unusual cytogenetics of our patient inspired this literature search, which found no previously published systematic reviews describing the cytogenetics and phenotypic characteristics of unbalanced t(9;22) cases.

Though balanced translocations are common, occurring in at least 1 in 500 individuals, our patient’s constitutional balanced t(9;22)(q34;q11.2) is rare (Gardner, 2004). Her distinct translocation and abnormal metaphase FISH finding, albeit without gene fusion of *BCR-ABL1*, could have led to a misdiagnosis of CML if she had also presented with leukocytosis. Czuchlewski et al. (2011) reported a patient with constitutional t(9;22)(q34;11.2) and leukocytosis, which was differentiated from CML using FISH and the identification of the constitutional translocation in all cells examined. FISH demonstrated a juxtaposition of two intact probes rather than the fusion seen in the Philadelphia chromosome. Our patient’s translocation was also observed in all cells examined, indicating a similar germline translocation that mimics the Philadelphia chromosome.

Balanced t(9;22) carriers and their families should be counseled on the fertility implications and the reproductive risks of t(9;22) to help guide parents in their reproductive decision-making. Firstly, parents with a balanced t(9;22) have an increased risk of pregnancy loss. Though it is unclear if our patient’s previous pregnancy losses were related to her balanced translocation, we found in our review that most balanced t(9;22) carriers had at least one miscarriage, many of whom had recurrent pregnancy loss. Translocations of maternal origin carry an increased risk for recurrent pregnancy loss, while translocations of paternal origin increase the risk of male infertility (Griffin and Finch, 2005). In a cohort study of 132 Sri Lankan balanced translocation carriers, 63.6% experienced subfertility or recurrent pregnancy loss, likely due to an unbalanced translocation (Paththinige et al., 2019). These manifestations carry profound consequences for patients attempting family planning.

Secondly, balanced t(9;22) carriers should be counseled on their risk of having a child with an unbalanced translocation. Based on the breakpoints of our patient’s translocation, she has an increased likelihood of having a child with DiGeorge syndrome, 9q subtelomeric deletion syndrome, dual genetic diagnosis of both syndromes, or other unbalanced genomic outcomes (McGoey and Lacassie, 2009). A prospective trial in 2012 analyzed the reproductive outcomes of 54 balanced translocation carriers over two years and found that 12.5% had a subsequent pregnancy with the same or a different unbalanced translocation cytogenetically or phenotypically from their first affected pregnancy (Kochhar and Ghosh, 2013). Children who carry their parents’ balanced translocation also have a theoretical risk of transmitting an unbalanced translocation to their future offspring (Sanger et al., 2005). Balanced t(9;22) carriers are also at an increased risk of having a child with a spectrum of phenotypes associated with unbalanced t(9;22) cases, including cardiac abnormalities, neurological findings, intellectual disability, urogenital anomalies, respiratory or immune dysfunction, and facial or skeletal dysmorphias. However, our findings do suggest that unbalanced cases born to parents with balanced t(9;22) have a high rate of survival into adulthood in the absence of major cardiac abnormalities.

### Strengths and Limitations

This systematic literature review is the only comprehensive assessment of constitutional t(9;22) carriers and the outcomes and phenotypes of their unbalanced offspring. We also incorporated a case from our institution of a balanced t(9;22)(q34;q11.2), an analog of the somatic balanced translocation seen in the Philadelphia chromosome of CML. There are only two other published cases of a patient with balanced t(9;22)(q34;q11.2) (McGoey and Lacassie, 2009; Czuchlewski et al., 2011). The rarity of such a translocation underscores the importance of publishing similar cases.

A limitation of this review is the inclusion of clinical cases of t(9;22) with various chromosomal breakpoints, each of which are associated with a range of possible phenotypic outcomes. There is a relatively small number of accrued cases and each paper reported their cases with a varying level of detail. Additionally, five of the 15 balanced translocation carriers included in the review did not have the karyotype results for each of their offspring reported, limiting insight into the cytogenetic variability amongst siblings (Blank et al., 1975; Tihy et al., 2000; El-Fouly et al., 1991; Sanger et al., 2005; Bouhjar et al., 2011). Reported family histories of balanced translocation carriers and history of prior miscarriages were also sparse. Given the known propensity of balanced t(9;22) for familial recurrence and recurrent pregnancy losses, a lack of reporting limits our study’s ability to review the significance of these correlations in this population.

## Conclusion

Our review provides a cytogenetic breakpoint-based genotype-phenotype correlation for unbalanced cases of parental constitutional reciprocal translocation (9;22) carriers. This literature review helps providers give anticipatory guidance to balanced t(9;22) carriers on potential pregnancy outcomes. Balanced t(9;22) carriers should be counseled on the increased risk of recurrent pregnancy loss, subfertility, and having a child with an unbalanced translocation that could lead to an array of phenotypic manifestations. Phenotypic findings in unbalanced cases include cardiac abnormalities, neurological findings, intellectual disability, urogenital anomalies, respiratory or immune dysfunction, and facial or skeletal dysmorphias. Genetic counseling is recommended for any balanced translocation carrier or breakpoints regardless of the chromosomes that are involved.

## Data Availability

The original contributions presented in the study are included in the article/Supplementary Material, further inquiries can be directed to the corresponding author.

## Appendix A

**Database: PubMed**

Results: 45

Search: (“(9;22)” AND translocation AND (congenital OR constitutional OR inherited))

**Database: Scopus**

Results: 52

Search: TITLE-ABS-KEY(“9;22”) AND TITLE-ABS-KEY(translocation) AND TITLE-ABS-KEY((congenital OR constitutional OR inherited))

## Appendix B

**Methods for Cytogenetic Analysis, FISH, and CMA for Our Case Report**

*Conventional Cytogenetic Analysis for Our Case Report*

Peripheral blood lymphocytes were cultured in PB-MAX™ medium supplemented with stimulant phytohemagglutinin (PHA) for 72 h. Metaphase chromosomes for Giemsa banding pattern by trypsin digestion with Wright stain (GTW banding) were prepared according to standard procedures. Metaphases were karyotyped with GenASI Cytogenetics Analysis System (Applied Spectral Imaging, Carlsbad, CA), and karyograms were described according to the International System for Human Cytogenetic Nomenclature 2021. Coordinates of cells with chromosome aberrations were documented, and the slides were subsequently prepared for metaphase FISH analysis.*Interphase and Metaphase FISH (Fluorescence in situ Hybridization) Assays for Our Case Report*

Interphase FISH was undertaken using BCR/ABL1/ASS1 Tri-Color DF FISH Probe (Vysis, Abbott Park, IL). Metaphase FISH was performed on the de-stained GTW slides. Standard FISH protocol was followed. FISH images were captured and analyzed with a GenASI FISHView Analysis System (Applied Spectral Imaging, Carlsbad, CA).*Chromosome Microarray Analysis for Our Case Report*

Single nucleotide polymorphisms (SNP) microarray analysis was performed using the Affymetrix Cytoscan HD platform which uses over 743,000 SNP probes and 1,953,000 NPCN probes with a median spacing of 0.88 kb. 250ng of total genomic DNA extracted from lymphocytes was digested with Nspl and then ligated to Nspl adaptors, respectively, and amplified using Titanium Taq with a GeneAmp PCR System 9700. PCR products were purified using AMPure beads and quantified using NanoDrop 8000. Purified DNA was fragmented and biotin labeled and hybridized to the Affymetric Cytosan HD GeneChip. Data was analyzed using Chromosome Analysis Suite. The analysis is based on the GRCh37/hg19 assembly.
